# Shiitake mushroom-induced flagellate erythema

**DOI:** 10.1093/omcr/omaf293

**Published:** 2026-01-25

**Authors:** Dimitrios Karponis, Zoe C Venables

**Affiliations:** Norwich Medical School, University of East Anglia, Norwich, United Kingdom; Department of Dermatology, Norfolk and Norwich University Hospital, Norwich, Norfolk, NR4 7UY, United Kingdom; Norwich Medical School, University of East Anglia, Norwich, United Kingdom; Department of Dermatology, Norfolk and Norwich University Hospital, Norwich, Norfolk, NR4 7UY, United Kingdom; National Disease Registration Service, Data and Analytics, NHS England, United Kingdom

**Figure 1:**
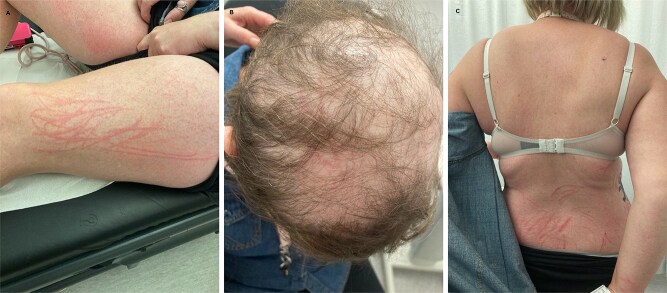
Flagellate dermatitis secondary to shiitake mushrooms on the left thigh (panel A), scalp (panel B) and lower back, with characteristic sparing of the upper back, which is out of reach for the patient (panel C).

A 38 year old female presented with generalised pruritus for 5 days followed by dermatographism and widespread, non-blanching, linear erythema for 2 days, starting on her thighs (Fig. 1A), involving the scalp (Fig. 1B) and sparing areas out of reach (Fig. 1C). Symptoms appeared 2 days after ingestion of stir-fried Shiitake mushrooms. Her background included chronic myeloid leukaemia treated with allogeneic stem cell transplant and tyrosine kinase inhibitors 10 years ago, and radiotherapy for atypical meningiomas. Of relevance to this presentation, there was no exposure to bleomycin, peplomycin or docetaxel. History and investigations (serum autoantibodies and myositis panels) excluded dermatomyositis, adult-onset Still’s disease and recent viral illness, other causes of flagellate erythema. Symptomatic treatment with topical betamethasone valerate and oral fexofenadine resulted in complete resolution of the rash and pruritus after 2 weeks. In summary, consumption of raw or undercooked Shiitake mushrooms is an important cause of flagellate dermatitis. This usually appears within 1–3 days and resolves within 3 weeks of consumption. Treatment is symptomatic with oral antihistamines and topical corticosteroids. It is entirely preventable by adequately heating the Shiitake mushrooms to at least 130–145°C, to inactivate the causative polysaccharide, lentinan.

